# Change of dominant material properties in laser perforation process with high-energy lasers up to 120 kilowatt

**DOI:** 10.1038/s41598-023-48511-9

**Published:** 2023-12-07

**Authors:** Stefan Reich, Marcel Goesmann, Dominic Heunoske, Sebastian Schäffer, Martin Lueck, Matthias Wickert, Jens Osterholz

**Affiliations:** Fraunhofer Institute for High-Speed Dynamics, Ernst-Mach-Institut, EMI, Ernst-Zermelo. 4, 79104 Freiburg, Germany

**Keywords:** Metals and alloys, Applied mathematics, Laser material processing

## Abstract

In laser materials processing, energy losses due to reflection, heat conduction and thermal radiation play an important role. In this publication, we show that with increasing laser intensity, the energy lost within the sample becomes less important for metal perforation processes. We compare the laser-matter interaction of aluminum and steel plates. Material parameters such as density, melting point and especially thermal conductivity differ strongly, leading to much longer perforation times for aluminum in comparison to steel at laser powers of 20 kW. However, this behavior changes at laser powers of more than 80 kW where the perforation times of aluminum become shorter than the corresponding times for steel. By comparing experimental data and simulations, we conclude that thermal conduction is the dominant factor of energy loss at low powers, but is reduced at high laser powers.

## Introduction

Continuous wave (cw) laser systems in the power range of 100 kW have been available for more than 10 years^1–5^. Such high laser powers cannot be achieved by a single laser. A combination of several lasers is required, for which different methods exist^6^. For single-mode lasers, coherent or spectral beam combination is usually used. For multi-mode fiber lasers, the passive fiber-based combination of fused fibers is common. To the best of our knowledge, the first industrial fiber laser with 100 kW was realized by IPG Photonics Corporation in 2013^2^.

In the field of laser material processing, small spot sizes are usually used^7^. Many common industrial applications such as cutting or welding metals use spot sizes below 1 mm, so the knowledge base is huge. However, there are applications where large spot sizes appear to be advantageous, such as laser welding of thick steel plates^8^. Furthermore, there are applications where lasers can be used as long distance effectors, e.g. in the field of security research^9–14^. Because large spot sizes require much higher laser powers, while maintaining similar intensities to those typically used for small spot sizes, large spot size applications could not be developed until the advent of such powerful lasers. As a result, there are only a few reports on applications using large spot sizes in the centimeter range^8,10–21^.

This literature on large spot laser-matter interaction covers many different aspects. There are reports on laser penetration of fiber reinforced plastics (FRP) showing strong interactions especially with the matrix material but also burning of the fibers^13,21^. Depending on the laser power and spot size, and therefore laser intensity, different penetration velocities and therefore perforation times are observed. As a final consequence, the compressive strength of FRP samples strongly decreases^21^. Environmental conditions such as tangential air flow also alter the penetration process leading to shorter perforation times in high Mach air flows^13^.

Unlike FRP specimens, airflow does not always reduce perforation times for metals. It depends more on the specific parameters. The air flow can lead to faster removal of molten material, but it can also lead to sufficient cooling of the specimen, which prolongs the perforation^13,16,17,22^. Melt pool dynamics can strongly depend on experimental conditions such as air flow velocity and direction of gravity^15,22,23^. Furthermore, a varying laser penetration is reported for nitrogen or normal air^15,24^. As the sample material heats up during laser irradiation, it reaches temperatures at which an oxidation process can efficiently occur, leading to an additional heat source. Such oxidation processes have been shown by spectroscopy to occur in steel and aluminum alloys^25^.

In addition to laser power, intensity also affects the perforation time. Higher intensities, due to smaller spot sizes, concentrate the energy on a smaller area. This means that a smaller amount of material has to be melted for the perforation, which reduces the perforation times^20,26^. However, reducing the spot size does not always reduce the perforation time. If a plasma is generated at the front side of the sample at a certain point due to the increasing intensities, such plasma can easily grow to a size of several tens of millimeters^27^. This may prevent the laser from reaching the sample, at least partially. Therefore, a minimum perforation time for spot diameters below 8 mm has been reported for iron samples^26^. Besides from reducing the processing efficiency, plasma can be useful for process control via plasma spectroscopy. From the plasma emission the temperature of the plasma can be calculated^27^, but also different material layers within the sample can be detected^25,28^.

For laser materials processing, it is not only important to have a laser source with sufficient power. The efficiency of the energy coupling also plays an important role. In addition to shadowing effects such as plasma or process plume in front of the sample (distances easily up to 10 cm), the absorptivity of the sample also plays a central role^15,17^. For metal samples of macroscopic thickness, the laser light can be either reflected (specular or diffuse) or absorbed as the transmission length is 9 nm for aluminum and 21 nm for iron^29^. Therefore, both reflectance and absorbance measurements are appropriate and will be used in the following. The physical reflectivity of pure materials such as aluminum and iron at room temperature at a wavelength of 1 $\upmu$m is 95.2% and 64.1%, respectively^29^. For aluminum, similar values have been reported for 1550 K when measured under vacuum^30^. However, this does not reflect the values observed in laser material processing^31^.

For aluminum, absorptivity values from 10% to 40% are reported^15,17,32–34^. On the one hand, it depends on the surface quality of the sample, where polished ones can have an absorptivity below 10%, while unpolished ones have values around 30%^33^. On the other hand, increasing values are reported above certain temperatures when measurements are made in air^15,33^. As this is not always observed^32^, this may also be influenced by the specific alloy composition.

For steel samples a similar behavior is observed but with a much stronger influence of the oxidizing atmosphere^26,27,32–35^. At room temperature, depending on the surface state, an absorptivity from 26% to 50% is reported. With increasing temperature the absorptivity increases due to oxidation^34,35^. Therefore, a strong increase in absorption is observed at about 600 $^{\circ }$C. If a mean value for the absorptivity is determined for the entire laser processing time, a dependence on the laser spot size is observed^26,27^. With increasing spot sizes also increasing absorptivity values are obtained. However, these are mostly mean absorptivity values which also take into account losses due to shielding effects in front of the sample nearby the sample surface, e.g. from a generated plasma.

The modeling and simulation of the laser-matter interaction is very complex^36^ and subject of current research. A large number of non-linearly coupled physical processes occur^7^. These processes take place on very different time and length scales. Numerical calculations for the entire process can therefore not be performed efficiently. A major focus of modeling is the identification of dominant sub-processes in a certain parameter regime and the reduction of the model equations to the most important effects or the dimensional reduction from 3D to 2D or 1D models^37^. In laser cutting, material removal can be caused by different processes. For cw-laser processing removal of the solid phase has been identified as the dominant process due to external gas jet driven material removal for laser cutting of metals^38^. For other materials like ceramics or thermosets, e.g., which are mostly processed by pulsed lasers also evaporation can be determinant.

When modeling laser perforation processes with beam radii in the range of a few centimeters, thermal aspects such as temperature-dependent heat capacity, conductivity and energy input are of particular importance. Perforation performance and its dependence on process and material parameters such as beam radius, absorptivity, thermal conductivity, specific heat capacity, vaporization temperature have been numerically investigated^23,26^. Numerical investigations of high-power laser experiments showed the influence of temperature dependent surface tension, gravity and vapor recoil pressure on perforation processes^18^. Aspects of melt pool behavior such as melt expulsion, Marangoni convection effects and the response to external forces through aerodynamic effects have been reported^15,39^. Depending on the laser spot parameters (small or large) and the application (cutting, welding or long range effectors), different parameters play an important role.

With the advent of high-power lasers with power levels in the range of 100 kW in continuous wave operation^3–5^, the question arises whether the knowledge of laser-matter interaction gained with lasers of a few tens of kilowatts power is still valid at such high power levels. So far, only results on laser welding with such high laser powers have been reported^4,40,41^. The high power available allowed welding depths of 70 mm for single-side welding and up to 125 mm for double-side welding^3,41^. On the side of the basic physical processes, however, nothing has been shown so far. Important scaling behavior in the laser-matter-interaction like a change in absorption or effects which lead to non-linear effects in the laser coupling efficiency are unknown until now.

In this paper we present the results of laser penetration experiments with a laser power up to 120 kW on aluminum and steel samples. With the extended power range compared to previous reports^14,20,26^ the available parameter range is extended. Besides the pure penetration process evaluation also an energy balancing is done. Two possible methods, a simple cylinder model and FEM simulations, are presented and compared for their applicability. By comparing the two energy balancing models, this study examines important aspects of laser processing with large spot sizes. From both models the effective absorptivity of the laser processing is calculated.

## Experimental setup

**Figure 1:**
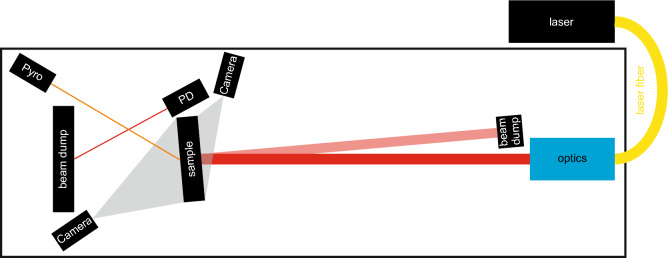
Experimental setup used for laser perforation tests. The laser beam was delivered by a fiber and shaped by an optics. The sample was imaged by optical and thermal cameras and two pyrometers (Pyro). To determine the perforation of the sample a photo diode (PD) detects laser light scattered from the beam dump behind the sample.

The experimental setup is similar to our previous publications^26,27^. However, the setup was upgraded with a laser of higher power and cameras of improved performance. An illustration of the setup can be found in Fig. 1.

The laser used was a ytterbium fiber-laser (YLS-120000, IPG Photonics Corporation, Germany) with a wavelength of 1070 nm and a maximum average power of 120 kW (1% power stability). It was operated in continuous wave mode. It consists of many separate laser modules which are spliced into a feeding fiber of 300 $\upmu$m and a process fiber of 600 $\upmu$m diameter. The process fiber has got a beam parameter product of 36 mm $\cdot$ mrad. At the output of the process fiber, a collimator (D85-F180 HP, IPG Photonics Corporation, Germany) determines the size and shape of the laser beam. At the sample position (5.64 m from the collimator) the laser beam is circular and had a diameter (D4$\upsigma$ of moment analysis^42^) of 35.0±0.3 mm with an almost Gaussian shape (super-Gaussian exponent 1.2). The local intensity (as mean value of 86% of the laser power within the determined beam diameter) was 0.09 kW/cm$^{2}$ for 1 kW up to 10.7 kW/cm$^{2}$ for 120 kW laser power. The laser power can be modulated up to 5 kHz. This corresponds to switching times of less than 100 $\upmu$s. Therefore, the ramp-up time of the laser power is not a significant factor in experiments where the experimental times are larger than one second.

The samples had a size of 200$\times$200$\times$10 mm$^{3}$ and were made of aluminum (1050A with 99.5% purity) and steel (DD11 with 99% iron purity). The plates were technical grade as provided by the industrial suppliers. The steel samples were cleaned with isopropanol to remove oil residues from the production process, the aluminum sample were supplied clean. The aluminum samples had a shiny surface with a roughness of $Ra={0.2}\, {\upmu }$m. The steel samples had a grayish surface typical for oxidized iron after annealing during manufacturing. The roughness was $Ra= {0.8}\, {\upmu }$m. The samples were positioned vertically with a horizontal impact of the laser.

Two color pyrometers (METIS M322 and H322, Sensortherm GmbH, Germany) were used to detect the backside temperature at the center of laser interaction (labeled as Pyro in Fig. 1). The M322 has got a measurement range from 600 $^{\circ }$C to 2300 $^{\circ }$C and was used for the steel samples, while the H322 was used for the aluminum samples and has got a measurement range from 400 $^{\circ }$C to 1200 $^{\circ }$C. As the emissivity of aluminum and steel is comparable low and not constant with temperature^43^, the back sites of the samples were painted with a thermally resistant paint (stable up to 800 $^\circ$C) (Dupli-Color Tuning Spezial-Lackspray Supertherm). The measured backside temperatures were used to determine the mean absorptivities from the simulations.

The perforation time was calculated from the trigger output of the laser and the increasing intensity behind the sample reflected from the beam dump and detected with a photo diode (labeled as PD in Fig. 1). The timing of the devices was realized with a delay generator (9524, Quantum Composers Inc., MT, USA). All the devices were triggered by the delay generator. The trigger signals and output voltage of the photo diode were recorded with a transient recorder (TransCom-CompactE-XL, MF Instruments GmbH, Germany).

## Numerical modeling

### Analytical modeling for energy balancing

For the energy balancing of the penetration process in section "Results and discussion" an analytical modeling is used, thereafter called the cylinder model. In this model the perforation hole is assumed as a cylinder with a diameter equal to the laser spot size $D4\sigma$ and a height equal to the sample thickness. With the material parameters (see Table SI1 in the supporting information) the energy $E_{cm}$ required for a complete heating up and melting of this cylinder is calculated by:

1 $$\begin{aligned} E_{cm} = c \cdot m \cdot \Delta T + H_{fus} \cdot m, \end{aligned}$$

with *c* as heat capacity, *m* as the mass of the cylinder, $\Delta T$ the temperature difference between room temperature and the melting temperature of the sample and $H_{fus}$ the enthalpy of fusion. To calculate the time for a complete homogeneous heating and melting of the cylinder, $t_{cm}$, $E_{cm}$ has to be divided by the laser power $P_{Laser}$. Since in the cylinder model only the material within the spot diameter $D4\sigma$ is used, also the power used for processing must be reduced to 86% of $P_{Laser}$, since this is the amount of power within $D4\sigma$. This leads to:

2 $$\begin{aligned} t_{cm} = \frac{E_{cm}}{0.86 \cdot P_{Laser}}. \end{aligned}$$

With that finally the effective energy coupling $\alpha _{cm}$ derived by the cylinder model can be calculated by:

3 $$\begin{aligned} \alpha _{cm} = \frac{t_{cm}}{t_p}, \end{aligned}$$

with $t_p$ being the experimentally determined perforation time.

### FEM simulation setup

As indicated in the introduction, the numerical simulations of the experiments carried out serve to gain a deeper understanding of the processes that are not directly measurable during the intense laser irradiation. In particular, the degree of absorption, more precisely the proportion of the laser energy introduced into the sample as heat, is a parameter of interest that has a large number of dependencies and uncertainties. Horak et al.^26^ presented a method that summarized these dependencies and effects in the so-called effective absorptivity $\alpha _{sim}$. For better comparability, the simulations presented here were performed using the same theoretical approach, but with a modified numerical setup to provide a more general framework and better performance of the simulations. For this reason, the numerical model for calculating the temperature distribution $T\equiv T(\vec x,t)\in {\mathbb {R}}$ with $\vec x\in \Omega \subset {\mathbb {R}}^3, t\in {\mathbb {R}}^+$ that develops over time inside the solid metal sample is largely based on the model presented in Ref.^26^.

Consider the time dependent non-linear heat equation

4 $$\begin{aligned} \frac{\partial T}{\partial t} = \frac{1}{\rho (T)c(T)}\nabla (k(T) \nabla T), \qquad \forall (\vec x,t) \in \Omega \times {\mathbb {R}}^+, \end{aligned}$$

in the space corresponding to the sample (same size as the experiments) $\Omega \subset {\mathbb {R}}^3$, with temperature dependent density $\rho (T)$, specific heat capacity *c*(*T*) and thermal conductivity *k*(*T*) (here $\rho (T)$, *c*(*T*) and *k*(*T*) $\equiv$ const.). In addition to the corresponding condition on the boundary $\partial \Omega$ in the form of an energy input by the laser $I_{L}$, heat losses through convection *C* with convection heat transfer coefficient $h=$ 3.0 W/m$^{2}$K and radiation *R* with surface emissivity of $\epsilon =$ 90% to the enviro nment are modeled as a flow over the surface by the Neumann boundary conditions on $\partial \Omega$:

5 $$\begin{aligned} k(T(\vec x,t))\frac{\partial T(\vec x,t)}{\partial \vec n} = R(\vec x,t) + C(\vec x,t) - I_{L}(\vec x,t), \end{aligned}$$

with $\frac{\partial T(\vec x,t)}{\partial \vec n} = \nabla T \cdot \vec n(\vec x,t)$, where $\vec n(\vec x,t)$ is the outward normal vector.

The laser intensity $I_{L}$ is defined by

6 $$\begin{aligned} I_{\text {L}}(\vec x,t)&={\left\{ \begin{array}{ll} \alpha _{sim} \frac{2P}{\pi r^2} \exp \left\{ -2\frac{||\vec x- \vec x_0||_2^2}{r^2} \right\} , &{} ||\vec x-\vec x_0||_2\le r\\ 0 , &{} \text {otherwise} \end{array}\right. } \end{aligned}$$

corresponds to a Gaussian profile, where *P* is the laser power, $\vec x_0\in {\mathbb {R}}^3$ the laser spot center on the specimen and $||\cdot ||_2$ the Euclidean norm. The energy within a radius $r=D4\sigma / 2$ is applied for $\vec x \in \partial \Omega _I$, the so-called irradiation area $\partial \Omega _I\subset \partial \Omega$, otherwise it vanishes.

The thermal solver in the commercial finite element method (FEM) Code LS-DYNA by Livermore Software Technology Corporation (LSTC) was used, which was completed with mentioned boundary conditions, a mixed (Crank-Nicolson) time step discretization and isotropic thermal material properties (see Table S1 in the supporting information). In addition, the spatial discretization was carried out using a refined grid of tetrahedron elements, which provides a higher resolution in regions with larger temperature gradients, i.e. in the center of the laser spot (see Fig. SI1 in the supporting information). The edge sizes of the used mesh differ from 0.2 mm up to 3 mm. The laser irradiation boundary condition was implemented via a user subroutine. Due to benefits in performance through less complex calculations of spot projections, a laser-fixed frame of reference was chosen. Here, a corresponding energy input is calculated for each surface element. The energy input results from the location-dependent laser power on the surface element weighted with the selected effective absorptivity $\alpha _{sim} \in {\mathbb {R}}$.

The effective absorptivity $\alpha _{sim}$ in the laser boundary condition was determined using an automated optimization script. Multiple simulations were run until a selected temperature matched the temperature at the same time and position on the backside of the specimen as measured with the two-color pyrometers. For aluminum this fitting point was chosen to be the saddle point at the melting temperature around 660 $^\circ$C. For steel, the transit of 800 $^\circ$C was chosen because of the limited thermal resistance of the paint.

In addition, an erosion criterion was added to the numerical model to simulate the removal of material by outflow. Elements have been optically eroded during the simulation, when they have reached an appropriate temperature, which in this case is the melting temperature. This particularly depicts reality if the melt has a low surface tension and thus immediately flows out.

## Results and discussion

### Perforation time

**Figure 2:**
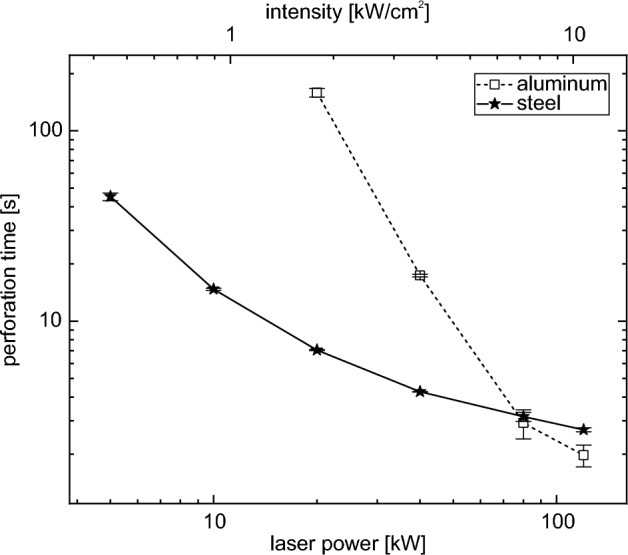
Double logarithmic plot of perforation time as a function of laser power (top axis: mean intensity of 86% within $D4\sigma$). The lines are only a guide to the eyes. While the perforation time of steel is shorter than that of aluminum at lower laser powers, this changes at high laser powers.

As expected, a decreasing perforation time is observed with increasing laser power, as shown in Fig. 2. Aluminum shows an almost linear behavior in this double logarithmic plot, which represents a power law in a linear plot. A power law fit ($y = a\cdot x^b$) of the perforation times up to 80 kW gives an exponent *b* of −2.88.

Both materials show a similar dependence of the perforation time on the laser power, despite the fact that they have very different material properties (see Table SI I in the supporting information). The comparison of the steel and aluminum samples shows that at lower laser powers the aluminum samples are perforated much slower than the steel samples. With the high laser power available in this work, we see that this changes. At around 80 kW, the perforation times are similar for both materials. Above this laser power, the aluminum samples are perforated faster than the steel samples.

This behavior of perforation time with increasing laser power is due to the different material properties (see Table SI I in the supporting information). The melting point of aluminum is almost 900 K lower than that of steel. However, the other thermal material parameters are higher for aluminum than for steel. The enthalpies of fusion and evaporation are slightly higher, while the thermal conductivity and specific heat capacity, especially in the liquid phase, are significantly higher. In summary, the energy required to melt a given volume of the aluminum sample starting from room temperature is only 35% of that required for steel, 2.6 J/mm$^{3}$ and 7.3 J/mm$^{3}$ respectively. From this simple comparison, which neglects thermal conductivity, one would expect the aluminum samples to be perforated faster than the steel samples. But especially at low laser powers, where perforation takes a long time, the thermal conductivity cannot be neglected^15^. We conclude that the longer perforation times for the aluminum samples at low laser powers must be attributed to significant lateral energy losses due to thermal conductivity. With increasing laser power the thermal conductivity becomes less dominant due to the fast material processing, which is why above about 80 kW the aluminum samples are perforated faster than the steel samples.

### Energy balancing

An important metric in evaluating the laser-matter interaction in laser processing is how much of the laser energy is used directly for processing and how much is lost for the intended process due to conversion to unintended forms of energy. In the following, the term energy loss is used to express the fact that the energy is no longer available for the intended process. For this purpose, an energy balancing is required. The energy emitted by the laser ($E_{Laser}$) is converted into four parts:

7 $$\begin{aligned} E_{Laser} = E_{Path} + E_{Refl.} + E_{Plasma} + E_{Bulk} \quad. \end{aligned}$$

Here $E_{Path}$ stands for the absorption and scattering of laser light in the path between the laser optics and the target. This also incorporates absorption in process plume. $E_{Refl.}$ represents the amount of energy getting reflected at the sample surface. In front of the sample surface a plasma can be generated. This absorbs energy from the laser. However, the plasma itself can again contribute to the heating of the sample through reradiation. Therefore $E_{Plasma}$ denotes for the energy absorbed by the plasma and not reradiated to the sample. $E_{Bulk}$ describes the energy finally absorbed by the sample. It is important to note, that here $E_{Path}$ and $E_{Plasma}$ are separated explicitly. Hence, $E_{Path}$ does not include the absorption in the laser path originating from the plasma. For the scope of this work $E_{Path}$, $E_{Refl.}$, and $E_{Plasma}$ will always be summarized into one contribution of energy loss outside of the sample. Transmission through the sample is not important as the transmission lengths of metals are only in the nanometre range.

The energy absorbed by the sample $E_{Bulk}$ can be split into different contributions:

8 $$\begin{aligned} E_{Bulk} = E_{Heat} + E_{Melt} + E_{Rad.} + E_{Heat \ cond.} \quad . \end{aligned}$$

Here $E_{Heat}$ stand for the energy used for material heating, $E_{Melt}$ represents the energy required for material melting at the melting temperature without additional heating, $E_{Rad.}$ denotes for the energy leaving the sample through thermal radiation and $E_{Heat \ cond.}$ describes the energy flow within the sample by heat conduction.

Equations (7) and (8) show, that there are several processes which are not directly contributing to the heating and melting of the sample material. In front of the sample energy can get lost by absorption in the complete laser path ($E_{Path}$), absorption in the plasma which is located near the sample surface ($E_{Plasma}$) and by reflection at the sample surface ($E_{Refl.}$). The first depends on the experimental setup such as laser-sample distance, air turbulences in the laser path and humidity but is independent of the sample material. Therefore it is constant in the here performed experiments. The absorption in the plasma is more complex since it is intensity and material dependent. At laser powers above 80 kW the intensity of the laser light exceeds 7.2 kW/cm$^{2}$. In this intensity regime the formation of a plasma plume in front of the sample can be observed. The absorption of the laser light due to inverse Bremsstrahlung in the plasma can exceed significant values^44^. In general, the formation of a plasma is observed at lower laser powers for steel compared to aluminum. This can be explained by the energy required for evaporation of material. Both materials have got similar boiling temperatures. But the energy required to reach this point and for evaporation differs strongly (see TableSI1 in the supporting information). For aluminum much more energy is required to heat up and vaporize the same mass of material compared to iron. Therefore, at least for the almost pure samples used here, the earlier plasma occurrence at the steel compared to aluminum can be addressed to the thermal properties of the materials. Finally, also the reflectivity of the sample is strongly material, temperature and temporal dependent. The primary surface finish as well as oxidation processes can change the reflectivity. Furthermore, after material melting again a significant change in reflectivity can occur.

Inside the sample, heat conduction leads to a flow of energy outside the actual processing area. The thermal conductivity is a material property. It is 3.4 times higher for aluminum compared to iron (see TableSI1 in the supporting information). The thermal radiation is also a material dependent property expressed by the emissivity. It can be changed by coating like the thermal paint on the sample back side or by surface structuring.

We present two ways of energy balancing. The first approach is the analytical modeling of the sample perforation by the cylinder model described in section "Analytical modeling for energy balancing". In this model any energy losses due to radiation and thermal conduction ($E_{Rad.}$ and $E_{Heat \ cond.}$ in Eq. (8), respectively) are not taken into account. The effective energy coupling $\alpha _{cm}$ incorporates their contributions to the material processing. This leads to an underestimation of the real absorptivity by definition. To be clear, this is not a full representation of the reality, where especially thermal conduction plays an important role especially for long processing times. But this cylinder model is intended to be as simple as possible. And as shown in the following, there are applications, where this model gives satisfying results. The second way is by FEM simulations, where the absorption coefficient is modified so that the simulations match the temporal evolution of the experiments (see section "FEM simulation setup" for more details). In this approach all energy terms of Eqs. (7) and (8) are taken into account.

**Figure 3:**
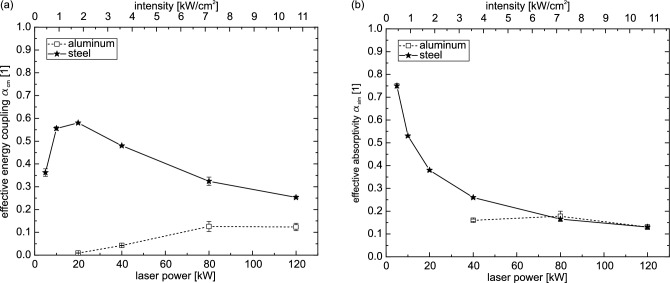
In (**a**) the effective energy coupling rate (perforations time experiment / perforation time cylinder model) calculated by a cylinder model and in (**b**) the effective absorptivity derived by simulations are shown for both materials. For steel a roughly decreasing and for aluminum a nearly constant behavior with laser power can be observed. For more details see the text.

In Fig. 3 the effective energy coupling $\alpha _{cm}$ calculated by the cylinder model is shown in (a) and the effective absorptivity $\alpha _{sim}$ derived by the FEM simulations is shown in (b). We assume that heat conduction is the most important loss mechanism. This is not considered in the cylinder model. Losses by heat conduction increase with process duration, hence the long perforation times in our case. This is especially true at low laser powers. Therefore, we attribute the decrease of the effective energy coupling $\alpha _{cm}$ for lower laser powers (for steel below 20 kW and for aluminum below 80 kW) in Fig. 3a to the heat conduction not considered in the cylinder model.

On the other hand, the final holes are mostly smaller than the spot diameter $D4\sigma$ of 35 mm (see Fig. SI3 in the supporting information). For aluminum, hole sizes were independent of laser power with diameters of around 30 mm. Furthermore, an almost constant diameter throughout the sample thickness is observed. However, the steel samples show a correlation between hole size and laser power. For low laser powers of 5 kW the hole diameters are around 15 mm, while for 120 kW the hole diameters increase to around 40 mm. These hole diameters are the front side diameters. Especially for the steel samples more conical holes are observed with significant smaller sizes at the back side. Therefore, even for the hole diameters of 40 mm for 120 kW laser power a mean hole size below the spot diameter is observed. Furthermore, it should be noted that the holes are larger in total than at the time of perforation because the laser was turned off with a slight delay after the first perforation of the sample. This means that the samples were irradiated a little longer (by less than 0.5 s) than the perforation time, which may have resulted in increased melting and flow out of material.

In the context of the cylinder model, smaller final hole sizes compared to the laser spot size results in an overestimation of the effective energy coupling $\alpha _{cm}$. This is because less material was heated and melted until perforation in reality than in the model. Consequently, the energy required to produce the resulting hole is less than that estimated by the cylinder model. Since for aluminum for all laser powers $\alpha _{cm}<\alpha _{sim}$ holds true, we conclude that this hole size effect only plays a minor role. Furthermore, it should be constant for all laser powers since the hole sizes and shapes are almost independent of the used laser power (see Fig. SI3 in the supporting information). For the steel samples, on the other hand, $\alpha _{cm}$ is larger than $\alpha _{sim}$ except of 5 kW laser power. This leads to the conclusion, that there the hole size effect might play a role. To investigate this effect further, an improved experimental setup with e.g. a 3D hole shape determination is required. Furthermore, the aim of the simple cylinder model was to only take as few parameters into account as required. From this point of view a hole shape measurement does not fit to the intention of the cylinder model used here.

The simulations, on the other hand, take into account thermal losses such as conduction and radiation. The simulations also produce a conical melt region, which is a better representation of the hole formed in the samples than the cylindrical model. However, any melt pool dynamics are not considered for the current simulation results, which are restricted to the dominant effect of heat conduction within the solid. An improvement over our previous publication^26^ is that artificial material removal can now be simulated by erosion of elements with a temperature higher than the melting temperature. This represents direct material removal after melting. This makes sense for most of the experiments here, since it can be observed for all steel samples and for the aluminum samples at high laser powers that the molten material flows out very quickly. For more details on the simulations see chapter "FEM simulation setup".

Figure 3b shows the effective absorptivity obtained from the simulations for steel and aluminum. A similar behavior to the cylinder model (Fig. 3a) with decreasing values for increasing laser powers is observed for the steel samples. As a consequence of the more complete representation of the overall process, there is no sudden decrease in the effective absorptivity below a 20 kW compared to the effective energy coupling of the cylinder model. This shows the superiority of the simulation over the cylinder model, especially in situations of low laser power and hence long processing times. Furthermore, for the steel samples above 10 kW, reduced values are obtained from the simulation compared to the cylinder model. At this laser power and below, we attribute the decreasing effective energy coupling of the cylinder model to the lack of energy loss due to thermal conductivity. Above this region of decreasing effective energy coupling, the fact of a lower final hole size compared to the cylinder model (as discussed above) becomes dominant, leading to higher values compared to the simulations.

Horak et al.^26^ used similar intensities of 0.7 kW/cm$^{2}$ to 13.7 kW/cm$^{2}$ as in this paper, but with a much lower laser power of only 2 kW and consequently significantly smaller spot sizes. An effective absorptivity between 30% and 80% was found for steel samples. The effective absorptivity was dependent on the spot size and hence the laser intensity. A similar behavior is observed in the present work. Again, a decreasing effective absorptivity is observed with increasing intensity, here realized by increasing laser power at fixed spot size. The values observed here are slightly lower than those reported by Horak et al.^26^. This could be due to the improved simulation setup, where the energy input is now more realistic due to a better implementation of the laser spot. Furthermore, it cannot be excluded that geometrical differences due to the changed size of the laser spot and the sample thickness have an influence on the resulting values. The changed steel alloy and surface properties such as roughness and oxidation state certainly also have an influence. Finally, the influence of the fit point at 800 $^{\circ }$C in the simulations (see section "FEM simulation setup") is also unclear.

Reported values of around 25% of effective energy coupling for experiments in inert gas atmosphere and increasing values up to 65% in oxidizing atmosphere^32,35^ do not reflect the experimental conditions of this paper. Here, almost pure iron was used which already had an oxidized surface compared to the polished stainless steel samples used by Hipp et al.^35^. For already oxidized steel samples, the absorptivity was reported to be around 80%^34^ with only small changes with temperature or irradiance (note, overall much lower intensities compared to those used here). Thus, the high energy coupling at low laser powers observed here is comparable to results previously reported for oxidized steel samples. Additional hemispherical reflectivity measurements with low laser powers (500 mW) performed on the samples used here showed a reflectivity of around 30%. This corresponds to an absorptivity of 70% which is in line with the values observed for low laser power processing. The decrease of the effective absorptivity for increasing laser powers observed here could be to some extent due to the change of the sample surface after melting. The original oxidized surface on the molten part of the sample flows out exposing a fresh unoxidized surface. The rate at which new oxidation might occur could not be evaluated and is beyond the scope of this work. We also attribute the decrease in energy coupling to some part to a shielding effect by emerging process products and the emergence of a plasma in front of the sample. Such stronger shielding effects are mainly located in the first 10 cm in front of the sample surface.

For the aluminum samples the simulations reveal an almost constant value of the effective absorptivity, similar to the effective energy coupling in the cylinder model. The value of about 15% is above^34^, similar to^32^ and below^15^ results already reported. However, these deviations are supposed to be attributed to the surface finish or the composition of the specific alloy. Hemispherical reflectivity measurements (laser power 500 mW) showed a value of around 90% reflectivity, which corresponds here to an absorptivity of 10%. This is in fair comparison to the calculated values in the laser processing. In contrast to the steel samples, the values from the simulations of aluminum are always higher than those from the cylinder model. This is the case not only in the laser power range where the cylinder model fails due to the high energy losses by heat conduction, but also above that. This leads to the conclusion that for aluminum the somewhat smaller hole sizes compared to steel (see Fig. SI3 in the supporting information) do not have a dominant effect. Again, the high thermal conductivity could lead to similar energy losses as a larger produced hole would have required.

Globally, a much higher effective energy coupling in the cylinder model is observed for the steel samples compared to the aluminum samples. The simulations also show that the effective absorptivity of steel is higher than that of aluminum up to a laser power of 40 kW. However, starting at 80 kW both materials exhibit a similar effective absorptivity . This reflects the fact that at high laser powers the aluminum samples have a shorter perforation time compared to the steel samples. The perforation times for aluminum are only about 60% compared to steel at 120 kW. To melt the same volume of sample material, as assumed in the cylinder model, aluminum requires only 36% of the energy to heat and melt the sample compared to steel. In summary, both observations, the similar effective absorptivity and the shorter perforation times of aluminum compared to steel, support significantly reduced energy losses within the samples at high laser powers.

### Volume and mass removal rate

**Figure 4:**
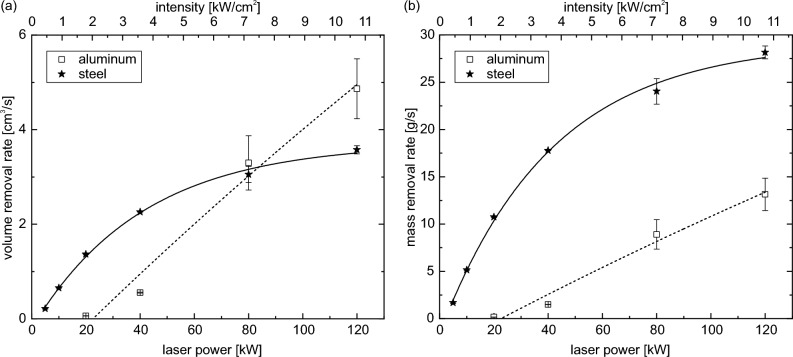
Volume removal rate (**a**) and mass removal rate (**b**) as function of laser power and intensity. The solid and dashed lines represent asymptotic fits. For steel an asymptotic behavior is observed. For aluminum a linear fit is equivalent to the asymptotic fit shown here. The error bars represent the standard deviations of the perforation times propagated to the removal rates.

In material processing with large laser spots, the volume removal rate or the mass removal rate is of interest, depending on the application. Both are shown in Fig. 4a and b respectively. The volume removal rate is calculated by dividing the volume of the previously used cylinder (sample thickness as height and $D4\sigma$ as diameter) by the perforation time. With increasing laser power, the removal rate increases asymptotically for steel samples and almost linearly for aluminum samples. The solid line represents an asymptotic fit to the data of the steel sample, which shows good agreement. The dashed line for the aluminum sample also represents an asymptotic fit, but a linear fit resulted in a comparably good agreement. Therefore, for the aluminum samples, it cannot be determined whether the behavior with laser power is linear or asymptotic. This difference may be due to the different mass outflow behavior of the two materials, as observed by surveillance cameras. Steel shows a more continuous outflow once the surface of the sample is molten. Aluminum, on the other hand, forms a molten bubble before flowing out^45^. This bubble formation tends to occur at lower laser powers and is not as dominant at the highest laser powers used here. However, the outflow process still differs significantly from that of the steel samples. While a saturation of the volume removal rate is observed for the steel samples at 3.9 cm$^{3}$/s, no saturation is evident for aluminum, at least up to the 120 kW laser power used. There may be saturation for much higher laser powers or intensities, but here we may still be in the region of nearly linear response within a globally asymptotic behavior.

As discussed before, in front of the sample a plasma can be generated. This can absorb significant amounts of laser power^44^. In general, the formation of this plasma starts at lower laser powers for steel compared to aluminum. Hence the plasma formation may contribute to the limited material removal rate for the steel samples.

Similar to the perforation time, the volume removal rate for the two materials also shows an intersection at around 80 kW. At lower laser powers, the volume removal rate of aluminum is lower than that of steel, whereas at high laser powers, the volume of aluminum is removed faster than that of steel. The data shown in Fig. 4 is based on the assumption of a cylindrical hole. As already mentioned (see Fig. SI3 in the supporting information), in reality smaller holes are observed for aluminum and rather conical holes are observed for steel. When these real hole volumes are taken into account, the values are slightly reduced. However, there are no significant changes.

Depending on the application, the mass removal rate may also be of interest. As the densities of aluminum and steel are very different, the results change significantly, as shown in Fig. 4b. Due to the much higher density of steel compared to aluminum, the overall mass removal rates of steel are much higher compared to aluminum. Even if the extrapolation shows an intersection, it is not certain that the extrapolation holds for such high powers.

## Conclusions

We have investigated the laser-matter interaction for cw laser radiation with powers up to 120 kW on aluminum and steel samples. We were able to show that the laser penetration processes in the two investigated materials differ in certain aspects. Aluminum has a much higher thermal conductivity than steel, leading to significantly higher energy losses at long processing times. This results in a much longer perforation time for aluminum compared to steel at low laser powers. At high laser powers, however, the much lower total energy required to heat and melt the same amount of material of aluminum dominates, resulting in a lower perforation time above roughly 80 kW.

To elucidate the effectiveness of the process, we compared the effective energy coupling derived from a cylinder model with the effective absorptivity obtained from FEM simulations. The cylinder model excludes many aspects such as thermal conductivity and radiation that are included in the simulations. Therefore, below a certain laser power, we found a strongly underestimated effective energy coupling, resulting in strongly decreasing values in the cylinder model. For high laser powers, however, a similar behavior to the simulations was observed. For aluminum, an almost constant value of above 10% is observed irrespective of the laser power. For the steel samples, on the other hand, the simulations revealed a decreasing effective absorptivity with increasing laser power from almost 80% down to above 10% again. In this case, the cylinder model overestimated the effective energy coupling to the non-cylindrical but more conical final holes.

This paper concentrates on large spot laser-matter-interaction. For the presented work a constant spot diameter of 35 mm was used. With a laser power of up to 120 kW the maximum realized intensity is almost 11 kW/cm$^{2}$. Future work can explore the question of a modified laser-matter interaction at higher intensities. With smaller spot sizes, higher intensities could be realized, which would address the open question of whether aluminum also shows an asymptotic behavior in volume and mass removal rate similar to steel at higher intensities. However, the simulations could also benefit from further improvements, such as the inclusion of any melt pool dynamics that are not currently considered. It is unclear whether and to what extent different dynamics correlate with changes in perforation times. For aluminum in particular, a high variance in perforation times suggests that different melt pool dynamics may be important.

### Supplementary Information

Supplementary Information.

## Data Availability

The data sets generated during and/or analyzed during the current study are available from the corresponding author on reasonable request.
