# Supplementary material for: Change of dominant material properties in laser perforation process with high-energy lasers up to 120 kilowatt
Source: Sci Rep. 2023 Dec 7;13:21611. doi: 10.1038/s41598-023-48511-9 (PMC10703938; doi:10.1038/s41598-023-48511-9)
Supplement: Supplementary file 1 — Supplementary Information. [file 41598_2023_48511_MOESM1_ESM.pdf]

Change of dominant material properties in laser perforation process with high-energy lasers up to 120 kilowatt

Stefan Reich[1,*], Marcel Goesmann[1], Dominic Heunoske[1], Sebastian Schäffer[1], Martin Lueck[1], Matthias Wickert[1], and Jens Osterholz[1]

[1]Fraunhofer Institute for High-Speed Dynamics, Ernst-Mach-Institut, EMI, Ernst-Zermelo. 4, 79104 Freiburg, Germany
[*]stefan.reich@emi.fraunhofer.de

Table SI 1. Material parameters for aluminum and iron used in the cylinder model and the simulations. Values from[1].

	aluminum	iron
density $[g/cm^3]$	2.7	7.87
absorption @ 1064 nm $[cm^-1]$	1092201	471909
reflectivity	0.9521 (1.2 eV)	0.641 (1.2 eV)
melting point $[°C]$	660	1538
enthalpy of fusion $[J/g]$	400	247
boiling point $[°C]$	2519	2861
enthalpy of vaporization $[J/g]$	10859	6214
thermal conductivity $[W/mK]$	237	80
thermal capacity (@25 $°C$) $[J/gK]$	0.904	0.449
thermal capacity (liquid) $[J/gK]$	0.6	0.16
energy consumption until molten $[J/g]$	971	929
energy consumption until molten $[J/cm^3]$	2621	7311

References

1. Lide, D. R. (ed.) *CRC Handbook Chemistry and Physics, 85th Edition* (CRC Press, 2004), 85 edn.

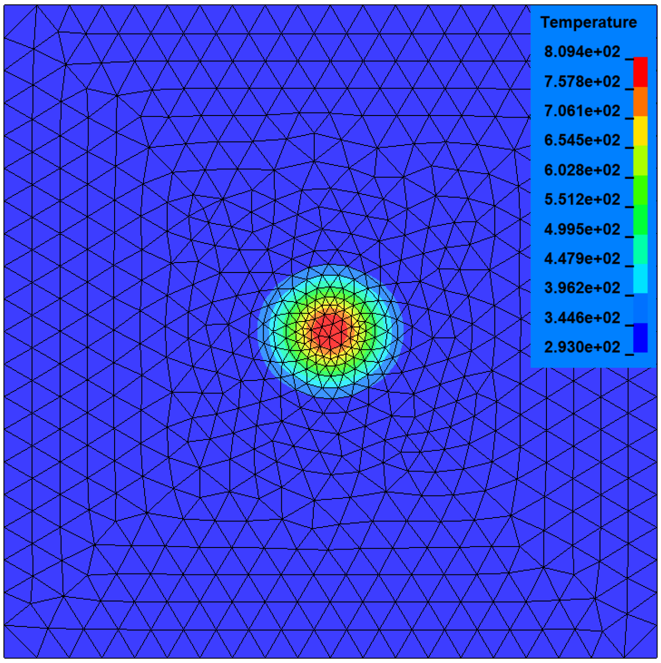

FIG. SI 1. Numerical discretization and visualization of a temperature distribution on the irradiation area.

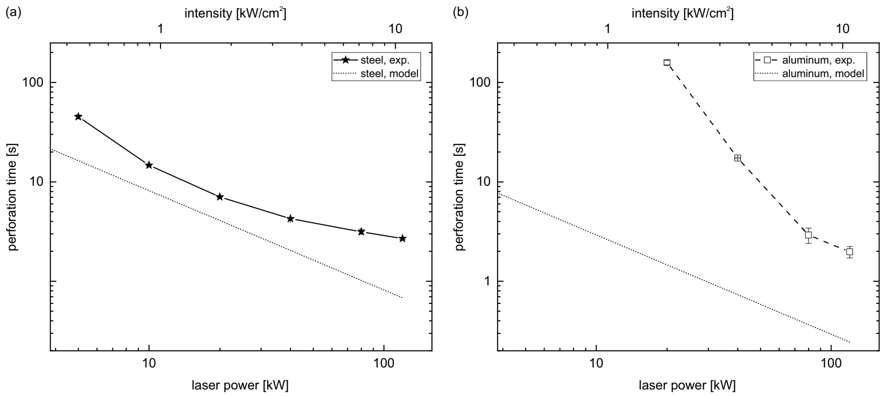

FIG. SI 2. Perforation time as function of the laser power plotted double logarithmic for steel (a) and aluminium (b) samples. The dotted lines represent calculations of the perforation time based on a molten cylinder of diameter $D4\sigma$. Used power input is 86 % of laser power and 100 % absorption of the sample is assumed. The calculated times are below the measured times showing directly the reduced absorption and energy losses such as radiation or heat conduction. The difference is larger for aluminum compared to steel. At aluminum samples, the holes are almost cylindrical while at the steel samples the holes are almost conical, hence the latter required a reduced amount of molten material compared to the cylinder estimation.

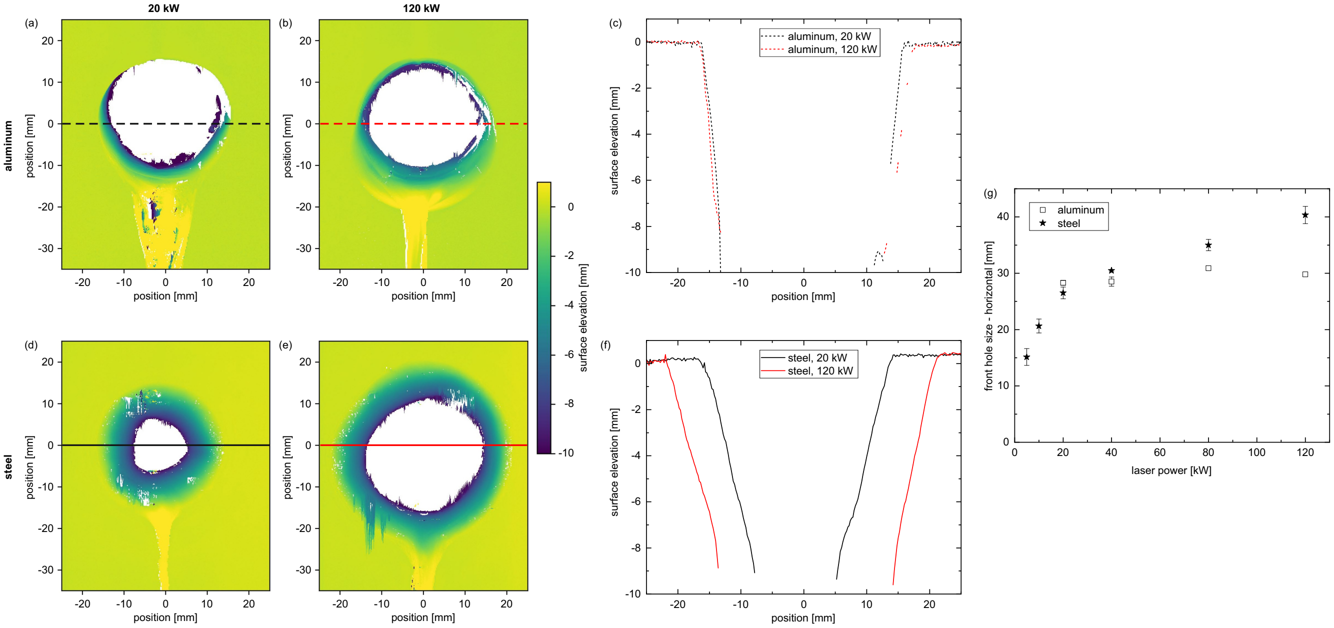

FIG. SI 3. Form of the resulting perforation holes measured with a triangulation setup for aluminum ((a) to (c)) and iron ((d) to (f)) for two representative laser powers. (c) and (f) show cross sections of the surface at vertical position 0. In (g) the hole horizontal size at the sample front side as function of laser power is shown. For aluminum similar holes with sharp edges are observed irrespective of the laser power. For steel strongly different hole sizes with conical shape are observed.
